# Editorial Comment: Megalourethra and urethrorectal fistula: a rare presentation and a challenging reconstruction

**DOI:** 10.1590/S1677-5538.IBJU.2015.0676.1

**Published:** 2017

**Authors:** Hubert Swana

**Affiliations:** 1 Pediatric Urology, Nemours Children’s Hospital Orlando, Orlando, FL, USA

Macedo et al. (1) present a video that elegantly demonstrates a one-stage repair for a child with a urethrorectal fistula along with a megalourethra. The anterior sagital trans-ano-rectal approach (ASTRA) was first described by Di Benedetto and Di Benedetto for use in clitoro-vaginoplasty (2). The ASTRA approach allows careful separation of the urethra and the rectum. It has proven to be useful in the treatment of urethral trauma, urogenital sinus anomalies, and urethral duplications (3). This video, when combined with the authors’ previous work, provides a useful reference to anyone planning to use this technique (4).

Hubert Swana, MD Pediatric Urology Nemours Children’s Hospital Orlando Orlando, FL, USA E-mail: hswana@nemours.org
